# *A novel mutation of the StAR* gene with congenital adrenal hyperplasia and its association with heterochromia iridis: a case report

**DOI:** 10.1186/s12902-019-0448-2

**Published:** 2019-10-30

**Authors:** Vera Splittstösser, Felix Schreiner, Bettina Gohlke, Maik Welzel, Paul-Martin Holterhus, Joachim Woelfle

**Affiliations:** 1 Pediatric Endocrinology Division Children’s Hospital, University of Bonn, Adenauerallee, 119 53113 Bonn, Germany; 2 Pediatric Endocrinology Division, Children’s Hospital, University of Kiel, Kiel, Germany; 3 Children’s Hospital, University of Erlangen, Erlangen, Germany

**Keywords:** StAR, Lipoid congenital adrenal hyperplasia, Heterochromia iridis

## Abstract

**Background:**

We report a novel mutation within the *StAR* gene, causing congenital adrenal hyperplasia, with the so far unreported association with heterochromia iridis.

**Case presentation:**

In a now 15-year-old girl (born at 41 + 6 weeks of gestation) adrenal failure was diagnosed in the neonatal period based on the clinical picture with spontaneous hypoglycaemia, hyponatremia and an extremely elevated concentration of ACTH (3381 pmol/l; ref. level 1,1–10,1 pmol/l), elevated renin (836 ng/l; ref. level 5–308 ng/l), and a decreased concentration of aldosterone (410 pmol/l; ref. level 886–3540 pmol/l). In addition to hyperpigmented skin the patient exhibited sectorial heterochromia iridis. Sequence analysis of the steroidogenic acute regulatory protein (*StAR*) gene showed a novel homozygous mutation (c.652G > A (p.Ala218Thr), which was predicted *in-silico* to be possibly damaging. Under daily steroid substitution her electrolyte levels are balanced while she became obese. Puberty occurred spontaneously.

**Conclusion:**

A novel mutation in the *StAR* gene was identified in a patient with severe adrenal hypoplasia and sectorial heterochromia iridis. We discuss a causal relationship between these two rare phenotypes, i.e. whether very high levels of ACTH and alpha-MSH during early development might have disturbed early differentiation and distribution of uveal melanocytes. If confirmed in additional cases, discolorization of the iris might be considered as an additional phenotypical feature in the differential diagnosis of congenital adrenal insufficiency.

## Background

The steroidogenic acute regulatory protein (StAR) is required for fast cholesterol transport to the inner mitochondrial membrane for steroidogenesis. Loss of StAR function is characterized by impaired synthesis of adrenal and gonadal steroids, causing adrenal insufficiency, disorder of sex development (DSD) and in some cases failure of pubertal development. Partial loss of StAR activity may cause the phenotypical picture of lipoid congenital adrenal hyperplasia (LCAH) or isolated adrenal insufficiency [1–5]. Prenatal steroid biosynthesis in placenta works independently of the presence of StAR protein. Postnatally, as StAR function is impaired or absent, some patients remain free of symptoms for the first 3 months of life. We report a novel *StAR* gene mutation (c.652G > A (p.Ala218Thr) in a girl with congenital adrenal insufficiency and heterochromia iridis.

## Case presentation

**Fig. 1:**
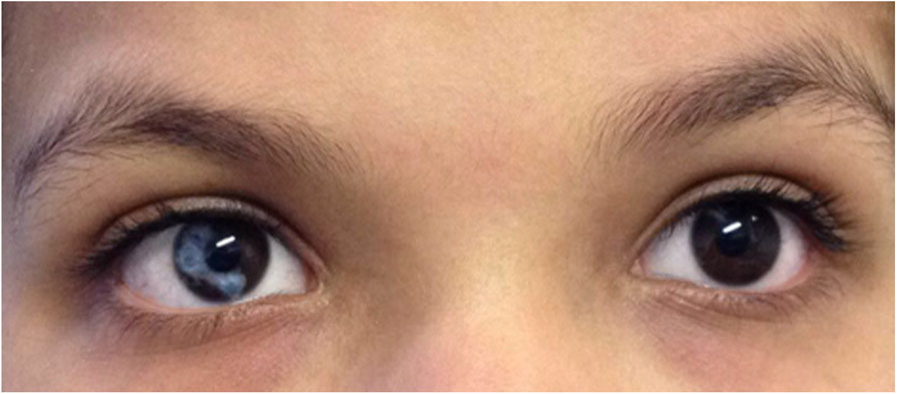
Heterochromia iridis with a brown- and blue-colored iris, putatively caused by significantly increased MSH production due to severe adrenal insufficiency and HPA axis activation

A term neonate (3760 g) with normal female external genitals was born as the second child of healthy consanguineous parents of Moroccan origin. 48 h after spontaneous delivery she presented with asymptomatic hypoglycaemia (blood glucose < 1 mmol/l) and was therefore treated with parenteral glucose application. During the following days she exhibited recurrent vomiting associated with hyponatraemia (125 mmol/l), hyperkalemia (8 mmol/l) and metabolic acidosis. Based on the biochemical constellation of an extremely elevated level of ACTH (341 pmol/l; ref. level 1,1–10,1 pmol/l), elevated renin (836 ng/l; ref. level 5–308 ng/l), an insufficiently low cortisol concentration of 162 nmol/l (58–475 nmol/l) and a decreased concentration of aldosterone (410 pmol/l; ref. level 886–3540 pmol/l) congenital adrenal insufficiency was diagnosed. Steroid substitution was started and the patient referred to our hospital. Clinically, the girl exhibited hyperpigmented skin in particular around the nipples, small labia, hands and lips. In addition, sectorial heterochromia iridis with a brown left eye and a sectorially mixed brown and blue right eye was noted (Fig. 1). Further investigations revealed a normal female karyotype (46, XX), a normal uterus and ovaries on abdominal ultrasound.

**Fig. 2:**
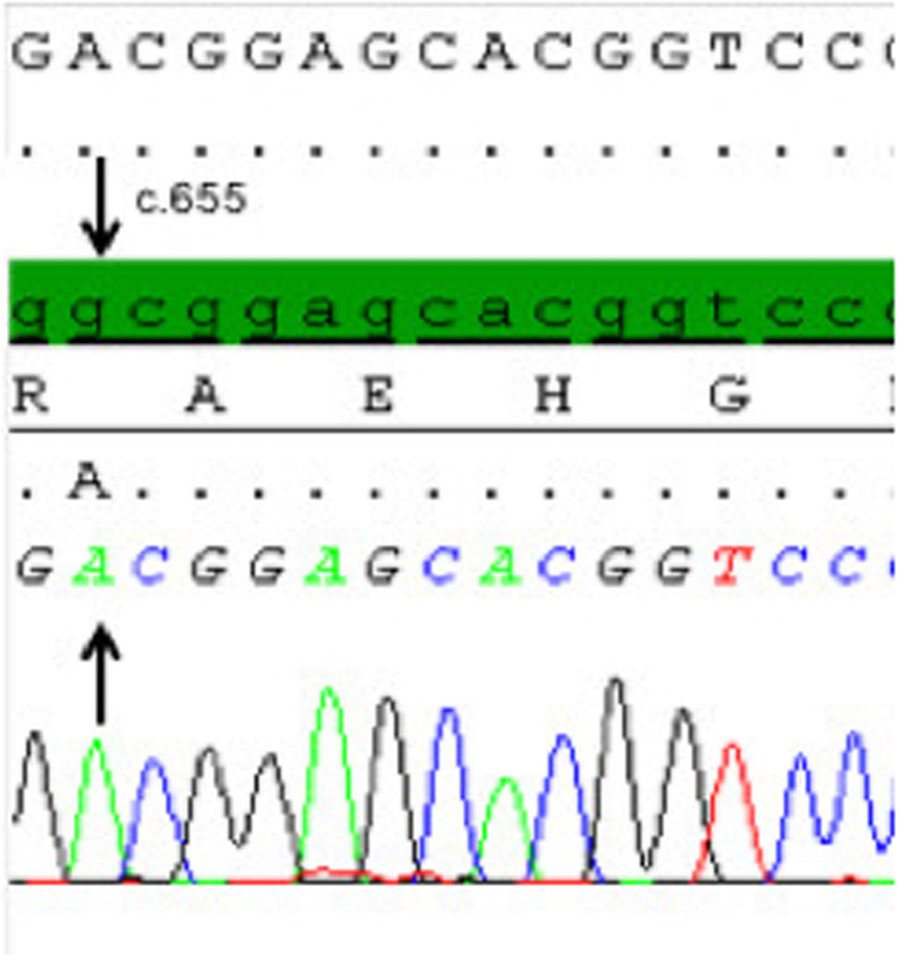
Sanger sequencing of the StAR gene: The wild type sequence is highlighted in green. The patient carries a homozygous G > A conversion at position c.652 in exon 6, as indicated by the arrow

Mutation analysis: Analysis of the *StAR* and *CYP11A1* gene was performed by polymerase chain reaction (PCR) amplification and direct Sanger sequencing of the coding exon and exon-intron-borders. Sequence analysis was performed in blood lymphocytes of the index patient and her mother. Sequence analysis of the *StAR* gene showed a so far non-described homozygous mutation in exon 6 of the *StAR* gene (c.652G > A; p.Ala218Thr) (Fig. 2). The healthy mother is a heterozygous carrier for the described mutation while the father and sister have not been tested yet. The mutation is predicted as probably damaging by PolyPhen (http://genetics.bwh.harvard.edu/pph2/).

The now 15 year old girl has been under regular attention in our pediatric endocrinology department since the neonatal period. Under 3-times daily hydrocortisone (9 mg/m^2^/day) and fludrocortisone (0,2 mg/day) substitution her electrolyte levels are balanced while the ACTH concentration remains elevated for a significant part of the day (4–688 pmol/l; ref. level 1,1–10,1 pmol/l). In particular during febrile infections in infancy her metabolic situation frequently deteriorated, sometimes associated with hypoglycemic/hyponatremic seizures.

The patient is growing within her target height range, while her bone age is delayed by 2 years. Within the last years she became increasingly obese (weight 97. percentile), thus she was treated with the lowest possible hydrocortisone dosage. At the age of 12 years, spontaneous thelarche occurred, associated with increasing serum estradiol levels, and age-appropriate concentrations of gonadotropins. Currently, at the age of 14.5 years, she is Tanner stage P1–2 B3–4 M1, with spontaneous menarche occurring at an age of 13 years and 11 months. Gonadotropin concentrations are still within the normal range (LH 6,3 U/l, FSH 3,6 U/L, estradiol 46 pg/ml), whilst androstendione and DHEAS concentrations are within a low to not detectable range.

## Discussion and conclusions

To the best of our knowledge, this is the first case report of a patient with adrenal insufficiency caused by a novel mutation within the *StAR* gene, who exhibits an association of a StAR defect in combination with sectorial heterochromia iridis. Heterochromia iridis, which can be further classified into a binocular and sectorial form, is a rare abnormality without influence on the eye’s function. In most affected individuals, it is observed already at birth, either isolated or as part of syndromic conditions such as Waardenburg syndrome, Horner’s syndrome, Sturge–Weber syndrome, Hermansky-Pudlak syndrome, hypomelanosis of Ito or linear scleroderma.

For both binocular and sectorial forms of isolated heterochromia iridis, familial occurrence resembling autosomal-dominant mode of inheritance has been described in various reports while other cases appeared sporadically. In addition, heterochromia iridis can occur in later life as a postinfectious consequence after chronic iridocyclitis. Furthermore, extensive iris naevus may be misinterpreted as sectorial heterochromia [6].

Human eye color depends primarily on the number of melanin granules (melanosomes) within superficial iris stromal melanocytes [7, 8]. Eye color inheritance is multifactorial, although the OCA2 melanosomal transmembrane protein seems to be a major determinant [9]. On a biochemical level, alpha-MSH levels are able to increase pigmentation and melanosome content [10, 11]. The total number of melanocytes in irides of blue-eyed, gray-eyed, and hazel-eyed healthy individuals does not differ [8]. However, in patients with Waardenburg syndrome, heterochromia iridis is considered to be due to a reduced number of both melanocytes and melanosomes in the bright part of the eye in comparison to the brown eye [12]. Due to the StAR defect with adrenal insufficiency and insufficient regulatory endocrine feedback regulation, our patient exhibited strongly elevated circulating levels of ACTH already during early development. Thus, one could speculate that very high levels of ACTH and alpha-MSH, which are cleaved from their common precursor molecule proopiomelanocortin (POMC), may have disturbed early differentiation and spatial distribution of uveal melanocytes finally resulting in sectorial heterochromia iridis.

As described above, we observed a normal pubertal course including spontaneous menarche in in our patient. This is in congruence with the few reported female StAR cases, in which (in contrast to male patients) spontaneous pubertal development was reported in many female patients [3]. Remarkably, to date first reports on pregnancies in females with StAR deficiency have been published [13].

To date, a more detailed analysis of the effect of this novel *StAR* gene mutation on the function of the STAR protein is lacking. Another mutation at the same position of the *StAR* gene (c.653G > A) was found in a previously described patient with adrenal insufficiency (p.Ala218Val). In expression studies using transfected Cos-1 cells, the ability of Ala218Val mutant cDNA regarding its ability to promote pregnenolone synthesis from endogenous cholesterol was analysed. For the Ala218Val mutation, a residual in vitro StAR reactivity of ≈20% was observed [3]. Considering the reported residual activity of this mutant, one could speculate that the described residual StAR activity would not completely impair gonadal sex steroid synthesis, which would explain the spontaneous puberty observed in our patient, harbouring a mutation within the same codon. However, codon 218 represents the first codon of exon 6 of the Star gene; thus, activity analyses using transfected p.Ala218V cDNA bear a certain risk for missing potential splicing abnormalities, so that unanswered questions remain regarding the functional in vivo consequence of this novel mutation.

To conclude, this case report is the first to describe a co-occurrence of severe adrenal insufficiency due to a StAR defect with sectorial heterochromia iridis. We speculate that very high levels of ACTH and alpha-MSH during early developmental stages may have disturbed early differentiation and spatial distribution of uveal melanocytes. Although we cannot clearly determine whether the association of severe adrenal insufficiency with sectorial iridial heterochromia is caused by this hypothetical mechanism or reflects only a coincidence of two very rare phenotypes, we suggest to include a structured analysis of iridal colorization in the small group of patients with congenital adrenal failure. If confirmed in additional cases, decolorization of the iris might be considered as an additional phenotypical feature in the differential diagnosis of congenital adrenal insufficiency.

## Data Availability

Not applicable (case report).

## Abbreviations

ACTH: Adrenocorticotropic hormone
DHEAS: Dehydroepiandrosterone sulfate
FSH: Follicle stimulating hormone
LCAH: Lipoid congenital adrenal hyperplasia
LH: Luteinizing hormone
MSH: Melanocyte-stimulating hormone
POMC: Proopiomelanocortin
StAR: Steroidogenic acute regulatory protein

## References

[1] Lin D, Sugawara T, Strauss JF, Clark BJ, Stocco DM, Saenger P, Rogol A, Miller WL (1995). Role of steroidogenic acute regulatory protein in adrenal and gonadal steroidogenesis. Science.

[2] Bose HS, Sugawara T, Strauss JF, Miller WL (1996). The pathophysiology and genetics of congenital lipoid adrenal hyperplasia. N Engl J Med.

[3] Nakae J, Tajima T, Sugawara T, Arakane F, Hanaki K, Hotsubo T, Igarashi N, Igarashi Y, Ishii T, Koda N, Kondo T, Kohno H, Nakagawa Y, Tachibana K, Takeshima Y, Tsubouchi K, Strauss JF, Fujieda K (1997). Analysis of the steroidogenic acute regulatory protein (StAR) gene in Japanese patients with congenital lipoid adrenal hyperplasia. Hum Mol Genet.

[4] Baker BY, Lin L, Kim CJ, Raza J, Smith CP, Miller WL, Achermann JC (2006). Nonclassic congenital lipoid adrenal hyperplasia: a new disorder of the steroidogenic acute regulatory protein with very late presentation and normal male genitalia. J Clin Endocrinol Metab.

[5] Bens S, Mohn A, Yüksel B, Kulle AE, Michalek M, Chiarelli F, Özbek MN, Leuschner I, Grötzinger J, Holterhus P, Riepe FG (2010). Congenital lipoid adrenal hyperplasia: functional characterization of three novel mutations in the STAR gene. J Clin Endocrinol Metab.

[6] Rennie IG (2012). Don’t it make my blue eyes brown: heterochromia and other abnormalities of the iris. Eye (Lond)..

[7] Imesch PD, Bindley CD, Khademian Z, Ladd B, Gangnon R, Albert DM, Wallow IH (1996). Melanocytes and iris color. Electron microscopic findings. Arch Ophthalmol.

[8] Albert DM, Green WR, Zimbric ML, Lo C, Gangnon RE, Hope KL, Gleiser J (2003). Trans Am Ophthalmol Soc.

[9] Sturm RA, Frudakis TN (2004). Eye color: portals into pigmentation genes and ancestry. Trends Genet.

[10] Sköld HN, Yngsell D, Mubashishir M, Wallin M (2015). Hormonal regulation of color change in eyes of a .cryptic fish. Biol Open.

[11] Smith-Thomas LC, Moustafa M, Dawson RA, Wagner M, Balafa C, Haycock JW, Krauss A, Woodward DF, Macneil S (2001). Cellular and hormonal regulation of pigmentation in human ocular melanocytes. Pigment Cell Res.

[12] Mullaney PB, Parsons MA, Weatherhead RG, Karcioglu ZA (1998). Clinical and morphological features of Waardenburg syndrome type II. Eye (Lond).

[13] Khoury K, Barbar E, Ainmelk Y, Ouellet A, Lehoux JG (2009). Gonadal function, first cases of pregnancy, and child delivery in a woman with lipoid congenital adrenal hyperplasia. J Clin Endocrinol Metab.

